# Zyxin Is Involved in Fibroblast Rigidity Sensing and Durotaxis

**DOI:** 10.3389/fcell.2021.735298

**Published:** 2021-11-18

**Authors:** Ai Kia Yip, Songjing Zhang, Lor Huai Chong, Elsie Cheruba, Jessie Yong Xing Woon, Theng Xuan Chua, Corinna Jie Hui Goh, Haibo Yang, Chor Yong Tay, Cheng-Gee Koh, Keng-Hwee Chiam

**Affiliations:** ^1^ Bioinformatics Institute A*STAR, Singapore, Singapore; ^2^ School of Biological Sciences, Nanyang Technological University Singapore, Singapore, Singapore; ^3^ School of Pharmacy, Monash University Malaysia, Subang Jaya, Malaysia; ^4^ Mechanobiology Institute, Singapore, Singapore; ^5^ School of Materials Science and Engineering, Nanyang Technological University, Singapore, Singapore; ^6^ Environmental Chemistry and Materials Centre, Nanyang Environment and Water Research Institute, Singapore, Singapore; ^7^ Energy Research Institute, Nanyang Technological University, Singapore, Singapore

**Keywords:** zyxin, focal adhesion, durotaxis, rigidity sensing, mechanotransduction

## Abstract

Focal adhesions (FAs) are specialized structures that enable cells to sense their extracellular matrix rigidity and transmit these signals to the interior of the cells, bringing about actin cytoskeleton reorganization, FA maturation, and cell migration. It is known that cells migrate towards regions of higher substrate rigidity, a phenomenon known as durotaxis. However, the underlying molecular mechanism of durotaxis and how different proteins in the FA are involved remain unclear. Zyxin is a component of the FA that has been implicated in connecting the actin cytoskeleton to the FA. We have found that knocking down zyxin impaired NIH3T3 fibroblast’s ability to sense and respond to changes in extracellular matrix in terms of their FA sizes, cell traction stress magnitudes and F-actin organization. Cell migration speed of zyxin knockdown fibroblasts was also independent of the underlying substrate rigidity, unlike wild type fibroblasts which migrated fastest at an intermediate substrate rigidity of 14 kPa. Wild type fibroblasts exhibited durotaxis by migrating toward regions of increasing substrate rigidity on polyacrylamide gels with substrate rigidity gradient, while zyxin knockdown fibroblasts did not exhibit durotaxis. Therefore, we propose zyxin as an essential protein that is required for rigidity sensing and durotaxis through modulating FA sizes, cell traction stress and F-actin organization.

## Introduction

Focal adhesions (FAs) play an important role in sensing mechanical cues in the extracellular matrix and transducing forces from the extracellular matrix into biological signals (Riveline et al., 2001). The cells can sense and respond to changes in the rigidity of the underlying substrates. When cells are grown on substrates of varying rigidity, they exert larger traction stress and migrate towards more rigid substrates in a phenomenon known as durotaxis (Lo et al., 2000). In order to sense substrate rigidity, the cells apply traction stress through FAs and actin stress fibres to measure mechanical responses of the substrate (Discher et al., 2005; Kobayashi and Sokabe, 2010; Prager-Khoutorsky et al., 2011). While the FA structure of mouse fibroblasts has been elucidated at the nanoscale level (Kanchanawong et al., 2010), little is known about the substrate rigidity sensing mechanisms of the cell.

Key proteins present in the FA in mouse fibroblasts include integrin, paxillin, focal adhesion kinase (FAK), talin, vinculin and zyxin (Kanchanawong et al., 2010). Some proteins in the FA including zyxin have been implicated to function as mechanotransducers, which transmit mechanical signals from the extracellular environment to the cytoskeleton of the cell (Wang et al., 1993). Zyxin was first discovered as a component of cell-substrate adhesions that also localized to the termini of actin stress fibres (Beckerle, 1986). It acts as a mechanotransducer to sense and relay mechanical signals to the cell (Hirata et al., 2008a). Zyxin also facilitates reinforcement of the actin cytoskeleton in response to mechanical forces (Yoshigi et al., 2005; Hirata et al., 2008b) and aids cell migration (Drees et al., 1999). It has also been reported that zyxin shuttles between the FAs and nucleus depending on mechanical stimuli in the extracellular environment of the cell (Beckerle, 1997; Nix and Beckerle, 1997; Yoshigi et al., 2005). Knocking down zyxin results in defects in actin cytoskeleton remodelling and reinforcement, reduces stress fibre and FA formation, as well as cancer cell migration and invasion (Harborth et al., 2001; Hoffman et al., 2006; Yamamura et al., 2013). While these studies clearly show that zyxin is involved in cell’s mechanotransduction pathway, the role of zyxin in substrate rigidity sensing still remains unknown. Since rigidity sensing is a key mechanism observed in various cell contexts including durotaxis, we aim to elucidate the role of zyxin, specifically in rigidity sensing which leads to directed cell migration through durotaxis.

In this study, we first determined the role of zyxin by knocking down zyxin in mouse embryonic fibroblasts, NIH3T3. We observed that silencing zyxin with siRNA led to changes in FA sizes, F-actin organization and cell traction stress magnitudes in NIH3T3 fibroblasts, resulting in impairment of the cells’ ability to sense and respond to changes in extracellular matrix rigidity. Cell migration speed of zyxin knockdown cells were also independent of the underlying substrate rigidity, unlike wild type NIH3T3 cells, which migrated fastest at an intermediate substrate rigidity of 14 kPa. Wild type NIH3T3 cells exhibited durotaxis (Lo et al., 2000) by migrating towards regions of increasing substrate rigidity when seeded on polyacrylamide gels with a gradient in substrate rigidity, while zyxin knockdown fibroblasts did not exhibit durotaxis. Therefore, we propose zyxin as an important molecule that is needed for durotaxis through modulating FA sizes, cell traction stress and F-actin organization.

## Materials and Methods

### Cell Culture

The cell lines used in this study were NIH3T3 (ATCC CRL-1658), MEF (ATCC SCRC-1040) and CT26.WT (ATCC CRL-2638). Dulbecco’s Modified Eagle Medium (DMEM) High Glucose (Invitrogen, CA, United States) with added supplements was used for cell culture. Cells were cultured in DMEM with 10% Fetal Bovine Serum (Invitrogen) and 1% penicillin-streptomycin combination antibiotic (Invitrogen). Cells were seeded at 80% confluence and transiently transfected with 75 nM pre-designed Stealth RNAi™ siRNA targeting mouse zyxin (Invitrogen, siRNA ID: MSS238956) using Lipofectamine 3,000 (Thermo Fisher Scientific) according to manufacturer’s instruction. To test zyxin knockdown efficiency, zyxin protein level was determined by western blot at 72 h.

### Western Blotting

Cells were washed in ice-cold PBS twice before being solubilised with ice cold RIPA buffer (Pierce) for 30 min. Lysates were centrifuged at 14,000 x g for 15 min at 4°C to pellet the cell debris. The supernatants were then mixed with 2x Laemmli sample buffer (Bio-Rad) and heated at 95°C for 5 min. The samples were then loaded on 10% sodium dodecyl sulfate polyacrylamide gel, separated via electrophoresis and transferred onto a nitrocellulose membrane. The blot was then incubated at room temperature for 1 h with 5% bovine serum albumin in Tris-buffered saline with 0.1% Tween (TBST) to block non-specific binding. Subsequently, the blot was incubated with antibodies specific for anti-vinculin (Sigma), anti-talin (Sigma) and anti-zyxin (Sigma) and β-actin (Santa Cruz), which were diluted with 5% bovine serum albumin in TBST, for 1 h at room temperature. β-Actin was used as a protein loading control. The blot was washed 3 times in TBST, for 5 min each, before and after incubating with a HRP-conjugated secondary antibody (Santa Cruz). The signal was then developed using Amersham ECL Prime Western Blotting Detection Reagent (GE Healthcare Life Sciences, Uppsala, Sweden) and imaged using the ChemiDocMP imaging system (Bio-Rad).

### Immunocytochemistry

Cells were cultured for 24 h on glass-bottomed dishes. Cells were then fixed with 4% formaldehyde (Sigma Adrich, MO, United States) for 20 min and permeabilized with 0.1% Triton-X (Sigma Aldrich) for 6 min at room temperature. After blocking with 2% bovine serum albumin (Sigma Adrich, MO, United States) for 1 h, primary antibody staining was performed for 2 h at room temperature. Focal adhesions (FAs) were stained using anti-mouse paxillin (Millipore, dilution 1:500), and anti-rabbit zyxin (Sigma, dilution: 1:800). Secondary antibody staining was performed for 1 h at room temperature, using Alexa Fluor 568 conjugated anti-mouse IgG antibody (dilution 1:1,000) and Alexa Fluor 488 conjugated anti-rabbit IgG antibody (dilution 1:1,000) from Invitrogen. Stress fibres were stained with Alexa Fluor 635 conjugated phalloidin (Invitrogen, dilution 1:100). Fixed samples were imaged using the Carl Zeiss LSM five LIVE inverted confocal microscope with a ×60 oil objective lens (Numerical Aperture 1.4) (Carl Zeiss Microscopy) at 12-bit.

### Image Analysis for Cell Area, FA Area, Colocalization of FA Proteins and Actin Alignment

The immunofluorescence images were processed and analysed using ImageJ. A 3D image stack of the fluorescently labelled cells were obtained. The cell area was quantified by segmenting the maximum projection of the stress fibre channel with a threshold value determined by the Otsu method, and a binary operation to fill holes was applied before obtaining the segmented cell area. To obtain the FA area, the paxillin staining channel was used. The imaging z-slice with the FAs in focus was manually selected for every cell and subjected to a 2 × 2 median filter. For the quantification, we included the high resolution images in Supplementary Figure S1, together with the segmented FA area. A fixed intensity threshold of 270 and FA size range of 0.2–5.0 µm (Lo et al., 2000) was applied to segment the FAs within the cell. The Pearson’s coefficient, which represents the extend of colocalization of zyxin and paxilin, was analysed using the *Coloc2* plugin of *ImageJ*, with the segmented FA as the mask within a whole cell. Before conducting the colocalization experiments, appropriate controls had been done to confirm that there was no bleed-through fluorescence between the 2 fluorescence channels. In the control experiments, the cells were immuno-stained for either zyxin or paxillin only, followed by both secondary antibodies, and imaged in both fluorescence channels. These experiments confirmed that fluorescence signal was only observed in the green (488 nm) channel when zyxin was stained and no signal was observed in the red (568 nm) channel, and vice versa for paxillin staining.

Similarly, actin alignment within the cell was quantified using the *OrientationJ* plugin in *ImageJ* after actin filaments had been segmented as regions of interest (ROI). Once ROI was selected, the alignment and coherency were measured. *OrientationJ* is an open source plugin’s package to automate the orientation analysis to calculate the actin stress fibre coherency within the cell.

### Traction Stress Analysis

For traction stress measurement, cells were seeded on polyacrylamide gels of Young’s modulus 6, 14, 31 and 60 kPa attached to glass coverslips. The polyacrylamide gels of various rigidities were fabricated following previously published protocols (Yip et al., 2013). The gels were functionalized with 50 μg/ml fibronectin diluted in HEPES buffer (0.5 M HEPES, pH 9.0) and cells were seeded on the fibronectin-coated gels for 24 h. Live imaging of the cells was done using the Carl Zeiss LSM five LIVE inverted confocal microscope with a ×60 oil objective lens (Numerical Aperture 1.4) (Carl Zeiss Microscopy). Details of the 2D traction stress calculations had been described in our previous work (Yip et al., 2013). Briefly, 2 sets of images of the fluorescent beads on the gel surface were obtained before and after cell detachment with 10% trypsin. The 2D displacement vectors, due to gel deformation brought about by the cell traction stresses, were determined by applying the digital image correlation algorithm, developed by Franck et al. (Maskarinec et al., 2009; Franck et al., 2011). After obtaining the displacement vectors, the stress tensor ε of the gel was found from the displacement-gradient technique and the material stress tensor σ can then be determined from the materials constitutive relation, σ = Eε/(1 + v) where E is the Young’s modulus of the gel and v is the Poisson’s ratio of the gel (v = 0.5).

### Fabrication of Gel Substrates

Preparation of polyacrylamide (PAA) gel was carried out by photopolymerization of 12% acrylamide and 0.52% bis-acrylamide (Bio-Rad), with a photoinitiator 1.5 mg/ml of Irgacure (Sigma Aldrich), to polymerize polyacrylamide gel upon UV exposure. Prior to the photopolymerization of PAA gel, the microscopic glass surface of the 4-well Chambered Coverglass (Thermo Scientific) were surface treated with 3-methacryloxypropytrimethoxysilane (Sigma Aldrich) to render a good adhesion between the PAA gel and the glass substrate. Then, 10 µL of acrylamide/bis solution was placed on the silanized microscopic glass and covered with an unsilanized coverslip (10 mmx5mm). The PAA gel was illuminated using an ultraviolet (UV) lamp (wavelength 285 nm, UVP Benchtop UV Transilluminators) for 1 min to initiate nucleation during polymerization reaction. It was then followed by uncovering the acrylamide/bis solution progressively by moving an opaque mask at a controlled speed of 16 um/s for 450 s using a syringe pump to obtain a stiffness gradient on PAA hydrogel. The resulting irradiation pattern created a hydrogel with Young’s modulus (E) gradient that changed from 17 ± 2 kPa in the most irradiated region to 2 kPa in the least irradiated one. After gel polymerization, the top glass coverslip was removed, and the gel was washed with deionized water thoroughly to remove unpolymerized and unreacted reactant for 24 h. The polyacrylamide gel was then further chemically treated with the N-Sulfosuccinimidyl-6-[4′-azido-2′-nitrophenylaimno] hexanoate (sulfo-SANPAH) under UV light irradiation for 15 min to activate the gel surface that will enable crosslinking with fibronectin (50 μg/ml). After the chemical treatment, residual sulfo-SANPAH on polyacrylamide gels was removed by washing with 50 mM HEPES buffer (pH 8.5) three times before coating with fibronectin.

### Characterization of Gel Rigidity

Rheological measurements of the PAA hydrogels were carried out using the MCR 501 rheometer (Anton Paar). Parallel-plate geometry was used. The upper plate is made of stainless steel with a 10 mm radius, while the bottom plate is the Teflon coated sample placement stage. The hydrogel samples were kept hydrated with PBS during the rheological experiments. The shear modulus (G*) was determined at a fixed shear strain of 0.5% over a frequency range of 0.1–3.72 Hz at room temperature to ensure a linear regime of oscillatory deformation. The Young’s modulus, *E*, was calculated from the measured average value of G* using the equation of E = 2G*(1 + ν), where the Poisson ratio, ν, is taken to be 0.45 for PAA hydrogel as described earlier (Yang et al., 2016; Yang et al., 2019).

### Cell Speed

The cells were plated sparsely on microscopic glass upon which a stiffness gradient gel coated with fibronectin had been formed. The cell nuclei were fluorescently labelled with 1 mg/ml Hoechst 34,580 (Invitrogen), to enable calculations of the cell migration speed. The cell migration speed was determined from time-lapsed images of the NIH3T3 cell nuclei, recorded every 30 min, over a period of 6 h. Images of the cell nuclei were subjected to a 3 × 3 median filter to remove noise and then segmented in ImageJ by applying a threshold value automatically determined by the “Triangle” method. The trajectories of the segmented cell nuclei were tracked and quantified using the MTrack2 plugin on ImageJ.

### Statistical Analysis

The 2-tailed Student’s t-test with unequal variance was performed in Excel. The means of two datasets are significantly different if the following *p*-values were obtained: *p* < 0.05 (*), *p* < 0.01 (**), *p* < 0.005 (***), or *p* < 0.001 (****).

## Results

### Zyxin Knockdown Resulted in Loss of Zyxin Localization at the FAs

**FIGURE 1:**
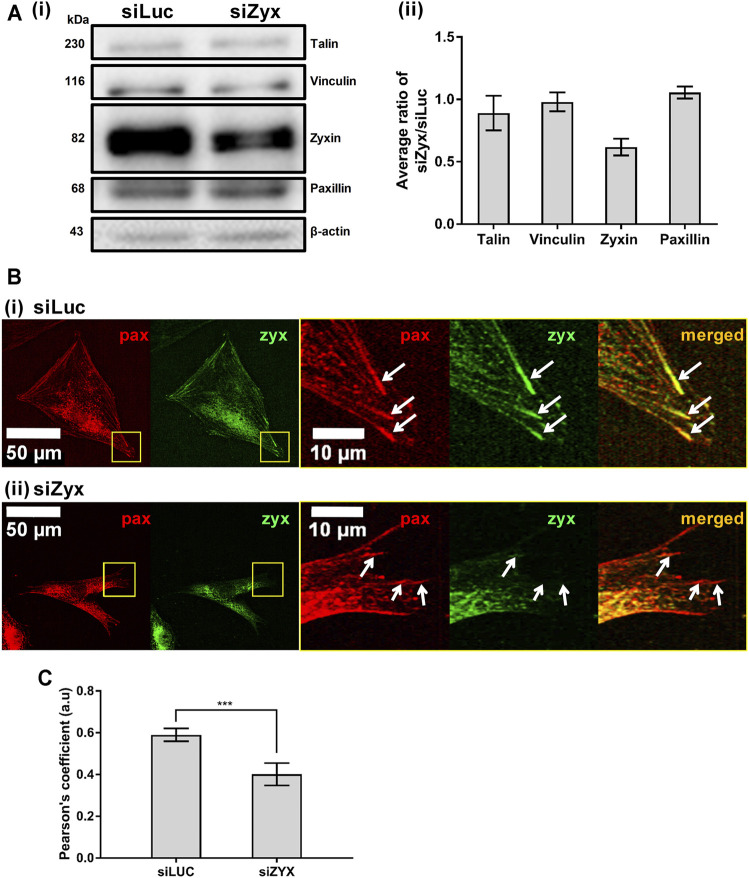
Knocking down zyxin in NIH3T3 cells does not affect levels of other FA proteins **(A)** 1) Western blot and 2) its quantification showing levels of zyxin in NIH3T3 cells transfected with a control siRNA (siLuc) and zyxin siRNA (siZyx). β-actin levels are used as loading control (n = 3) **(B)** immunofluorescence staining for paxillin (red) and zyxin (green) in 1) control siLuc-NIH3T3 and 2) zyxin knockdown siZyx-NIH3T3 cells on a stiff polyacrylamide gel (61 kPa). Right panels show the magnified view of the yellow boxes and the white arrows denote the FA stained by paxillin (red) **(C)** Quantification of zyxin colocalization with paxillin in siLuc-NIH3T3 and siZyx-NIH3T3 cells on the stiff polyacrylamide substrate (61 kPa). Pearson’s coefficient of 1 indicates perfect colocalization, 0 indicates no colocalization (n = 8). Error bars represent standard error of the mean. *** represents *p* < 0.005.

To investigate the role of zyxin in mouse embryonic fibroblasts, NIH3T3 cells were transfected with either the control siRNA (siLuc) or the zyxin siRNA (siZyx). We adopted lipofectamine 3,000 for siRNA transfection, to ensure superior transfection efficiency up to 79% in NIH/3T3 as reported from the datasheet of Thermo Fisher. Western blot was performed to confirm the knockdown efficiency of zyxin siRNA, siZyx (Figure 1A and Supplementary Figure S2A, D). Our result demonstrated that, up to 80% reduction on the total protein expression level could be achieved (Figure 1A and Supplementary Figures S2A, B). In addition, the statistical analysis shown in standard deviation and student t-test showed strong evidence that there were two different populations after the transfection (control group showed the zyxin expression population while the case group showed the zyxin deficiency population). We also confirmed that zyxin knockdown did not affect the levels of other key FA proteins (talin, vinculin and paxillin, Figure 1A). To examine the localization of zyxin at the FAs in both zyxin knockdown and zyxin expressing cells, we co-stained paxillin with zyxin. In this study, paxillin was chosen as the representative focal adhesion protein because paxillin has been reported to be a main component of FA in many studies (Kim and Wirtz, 2013; Hu et al., 2014; López-Colomé et al., 2017; Legerstee et al., 2019). It has also been reported that FAs stained by paxillin are similar in size and shape as those stained by vinculin, FAK and zyxin (Kim and Wirtz, 2013). By co-staining with paxillin, we confirmed that zyxin was absent at the FAs of the NIH3T3 cells transfect with siZyx (Figure 1B). Quantification of the colocalization of zyxin and paxillin also yield lower Pearson’s coefficient for the siZyx transfected cells (1 indicates perfect colocalization, while 0 indicates no colocalization), verifying that lesser zyxin localizes to the FAs of the zyxin-knockdown cells (Figure 1C).

### Zyxin Knockdown Impaired Substrate Rigidity-Mediated Cell Spreading and Growth of FA Size

Zyxin is known to be a mechanotransducer, we thus investigated if zyxin has a role in substrate sensing capacity. It has been reported that cells increase their spread area in response to increasing substrate rigidity as the size of mature FA increases (Solon et al., 2007; Yip et al., 2013). Since zyxin is a component of the FA, we measured and compared the spread area of NIH3T3 cells transfected with the control siLuc siRNA and siZyx siRNA on different polyacrylamide substrate rigidities.

**FIGURE 2:**
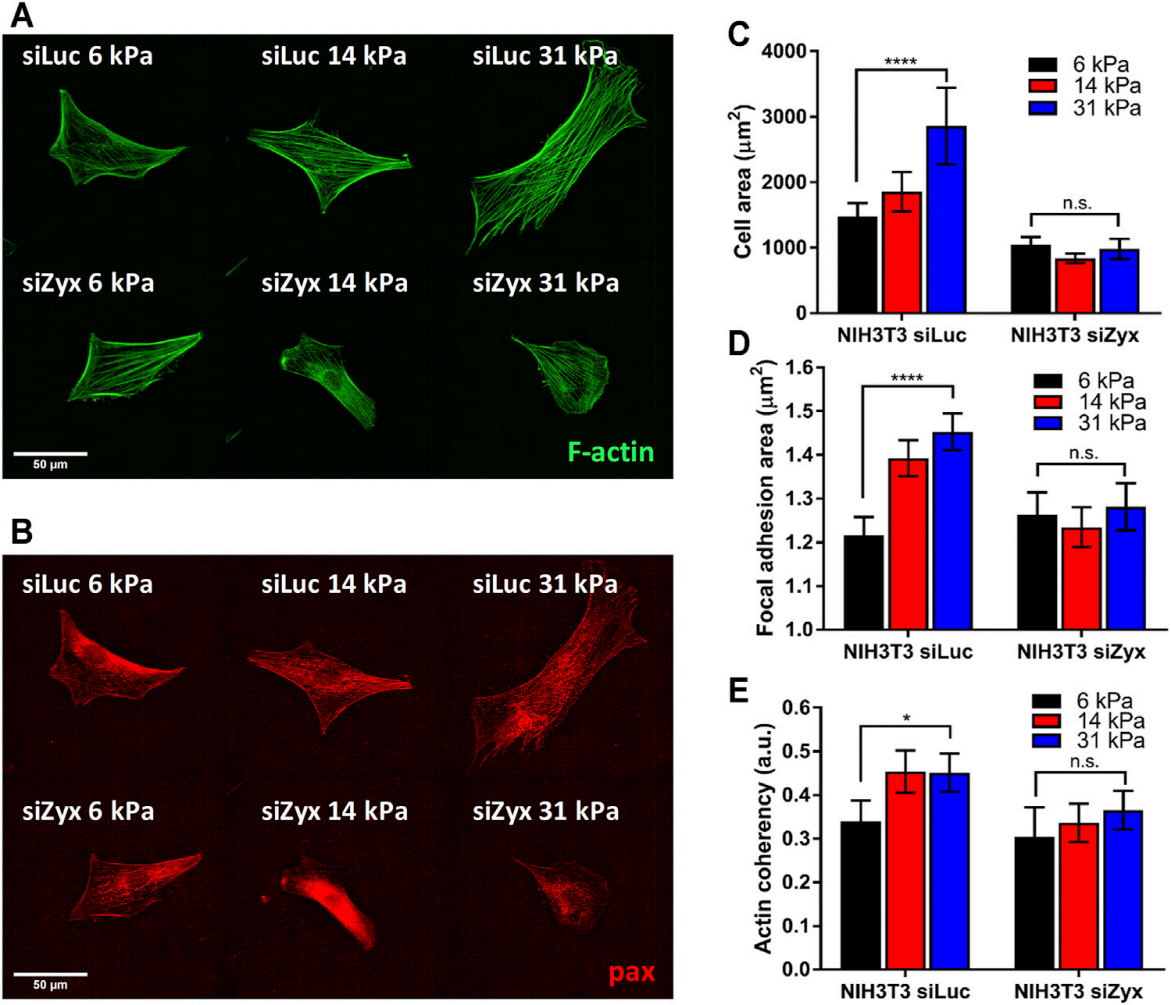
Zyxin-knockdown NIH3T3 cells do not show increased cell-substrate adhesion and actin polarization in response to increasing substrate rigidity **(A)** Representative F-actin (green) and **(B)** paxillin (red) immunofluorescence staining images for siLuc- and siZxy- NIH3T3 cells at different substrate rigidity **(C)** Graph of cell area vs substrate rigidity for siLuc- and siZyx- NIH3T3 cells **(D)** Graph of focal adhesion (FA) area vs substrate rigidity for siLuc- and siZyx- NIH3T3 cells **(E)** Graph of F-actin coherency vs substrate rigidity for siLuc- and siZyx- NIH3T3 cells. n = 12, 16, 19 for siLuc NIH3T3 6 kPa, 14 and 31 kPa respectively. n = 10, 15, 11 for siZyx NIH3T3 6 kPa, 14 and 31 kPa respectively. Error bars represent standard error of the mean. **** represents *p* < 0.001, * represents *p* < 0.05 and n.s represents not significant.

The absence of zyxin at the FA reduced substrate rigidity-mediated cell spreading (Figures 2A,B). We observed that NIH3T3 cells transfected with control siLuc showed larger cell spread area with increasing substrate rigidity (Figure 2C 6 kPa: cell area = 1,478 ± 199 µm; 31 kPa: cell area = 2,855 ± 583 µm). Unlike the siLuc transfected cells which doubled in spread areas, there was no significant increase in cell area with increasing substrate rigidity for siZyx transfected NIH3T3 cells (6 kPa: cell area = 1,040 ± 119 µm; 31 kPa: cell area = 980 ± 156 µm) (Figure 2C).

In addition, while the average area of individual segmented FA of control siLuc-NIH3T3 cells increased with increasing substrate rigidity (6 kPa: FA area = 1.22 ± 0.04 µm; 31 kPa: FA area = 1.45 ± 0.04 µm), there was no significant increase in the average area of individual segmented FA with increasing substrate rigidity for siZyx-NIH3T3 cells (6 kPa: FA area = 1.26 ± 0.05 µm; 31 kPa: FA area = 1.28 ± 0.05 µm) (Figure 2D). These results suggest that without zyxin localization at the FA, cells cannot respond to increasing substrate-rigidity by increasing cell-substrate adhesivity.

### Zyxin Knockdown Impaired Substrate Rigidity-Mediated F-Actin Alignment

Zyxin has been implicated in connecting the F-actin stress fibres to the FA complexes. When substrate rigidity increases, F-actin stress fibres become more aligned along the long axis of the cell (Prager-Khoutorsky et al., 2011; Trichet et al., 2012). We therefore investigated the organization of F-actin stress fibres in control and zyxin knockdown cells.

We observed that zyxin knockdown NIH3T3 cells had thinner stress fibres that are less aligned compared to control NIH3T3 cells (Figure 2E). In agreement with previous literatures (Prager-Khoutorsky et al., 2011; Trichet et al., 2012), the F-actin stress fibres in the control NIH3T3 cells become increasingly aligned with increased substrate rigidity as revealed by the coherency of the stress fibre staining (6 kPa: coherency = 0.34 ± 0.05, 31 kPa: coherency = 0.45 ± 0.04). However, in siZyx-NIH3T3 cells, the alignment of the stress fibres do not increase significantly with increased substrate rigidity (6 kPa: coherency = 0.30 ± 0.07, 31 kPa: coherency = 0.37 ± 0.04).

### Zyxin Knockdown Impaired Substrate Rigidity-Mediated Increase in Cell Traction Stress

It has been reported that cell traction stress increases with increasing substrate rigidity (Califano and Reinhart-King, 2010; Yip et al., 2013). In addition, durotaxis also involves the modulation of traction stress by substrate rigidity, with stiffer substrate eliciting stronger traction stress. Therefore, we also compared the average magnitude of traction stress exerted by zyxin-knockdown and zyxin-expressing cells on polyacrylamide substrates of differing rigidities, at 6 kPa, 14 and 31 kPa. This selected range was based on our previous finding (Yip et al., 2013), showing that fibroblasts exert traction stresses on substrates softer than 20 kPa to maintain constant strains. At rigidities beyond 20 kPa, stress through FA and actin fibres appears to be limiting as traction stress reaches a plateau (Yip et al., 2013). Furthermore, the fabrication of soft substrate lower than 6 kPa is very challenging because the gels become very soft and viscid, which affect cell adhesion. To our best knowledge, most studies either explored only a small range of rigidities (Califano and Reinhart-King, 2010), or only compared between two rigidity values (e.g., 14 and 30 kPa) (Lo et al., 2000). Therefore, we have selected a rigidity range of 6–31 kPa, to examine the rigidity sensing of zyxin in soft substrate (6 kPa) against an intermediate substrate (14 kPa) and stiff substrate (31 kPa).

Since the cell connects to the underlying substrate through the FA to exert traction stress for migration, and zyxin knockdown cells do not increase FA sizes significantly when substrate rigidity is increased, we hypothesized that knocking down zyxin would impair substrate rigidity-mediated increases in cell traction stress magnitudes.

**FIGURE 3:**
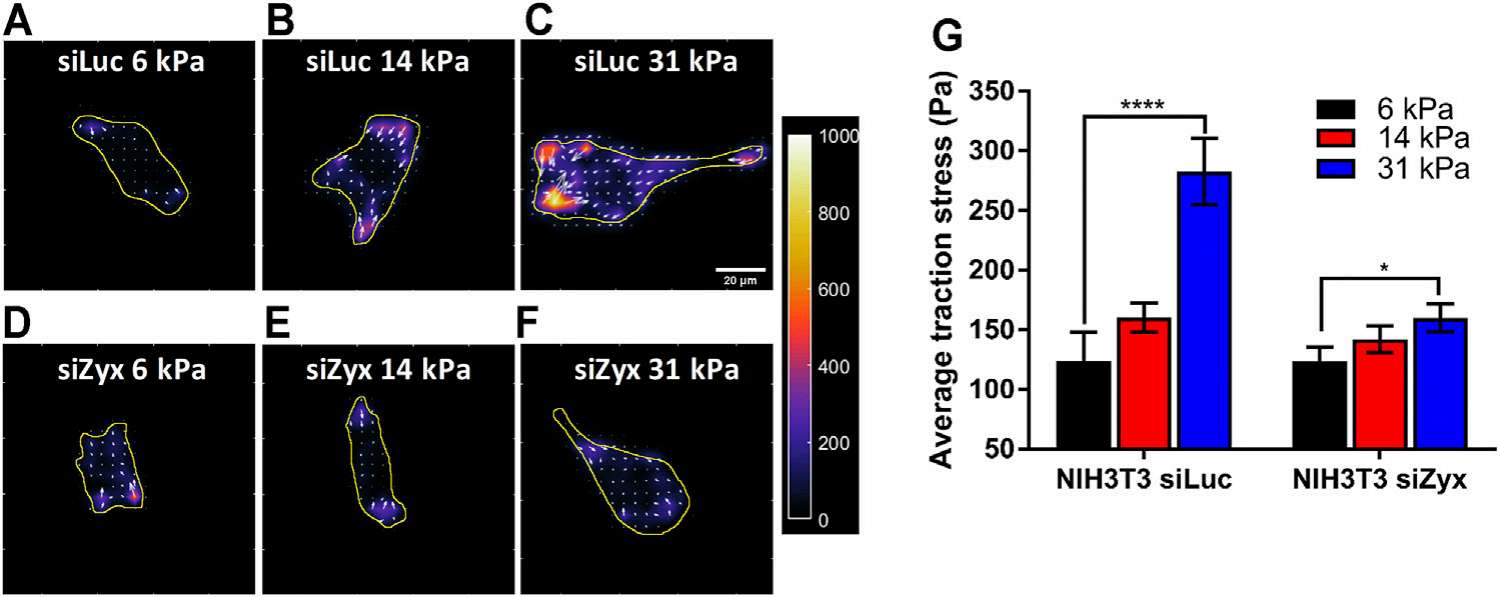
Zyxin-knockdown NIH3T3 cells do not increase cell traction stress in response to increasing substrate rigidity **(A–F)** Traction stress maps of **(A–C)** siLuc-NIH3T3 cells and **(D–F)** siZyx-NIH3T3 cells on substrates of rigidity 6 kPa, 14 and 31 kPa. Arrows denote the direction and magnitude of the traction stress in the x- and y-directions **(G)** Average traction stress magnitudes of siLuc- and siZyx- NIH3T3 cells on substrates of rigidity 6, 14, and 31 kPa, n = 11, 22, 19 for siLuc-NIH3T3 6 kPa, 14 and 31 kPa, respectively. n = 17, 25, 12 for siZyx-NIH3T3 6 kPa, 14 and 31 kPa, respectively. Error bars represent standard error of the mean. **** represents *p* < 0.001 and * represents *p* < 0.05.

Consistent with previous reports, we found that control siLuc-NIH3T3 cells (Califano and Reinhart-King, 2010; Yip et al., 2013) plated on substrates of differing rigidity (6 kPa, 14 and 31 kPa) exerted higher traction stress magnitudes on the more rigid 31 kPa substrates (Figures 3A–C, G) (average traction stress is 124 ± 24.0 Pa and 282 ± 28.0 Pa for siLuc-NIH3T3 on 6 and 31 kPa substrates, respectively). However, siZyx-NIH3T3 cells displayed less significant differences in average traction stress magnitudes when substrate rigidity was increased (Figures 3D–G) (average traction stress is 124 ± 12.0 Pa and 160 ± 11.6 Pa for siZyx-NIH3T3 cells on 6 and 31 kPa substrates, respectively).

The above observations were reproducible in different cell lines. We obtained similar results using a different mouse embryonic fibroblast cell line (MEF) and a mouse colorectal carcinoma cell line (CT26.WT) (Supplementary Figure S2). Control cells generated greater traction stress in response to increasing substrate rigidity. However, zyxin knockdown cells lost the ability to tune its traction stress in response to changes to the substrate rigidity. These findings suggest that zyxin knockdown leads to loss of substrate rigidity-mediated increase in cell traction stress, which in turn is important for rigidity sensing of substrates.

### Zyxin Is Required for the Sensing Substrate Rigidity During Cell Migration

Our observations thus far suggest that knocking down zyxin affected the ability of the cell to sense differences in substrate stiffness as rigidity-mediated increases in FA sizes, cell traction stress and actin alignment are impaired. FA sizes, cell traction stress magnitudes and stress fibre organization have been implicated in cell migration. Since cell migration speed is fastest at intermediate substrate rigidity (Peyton and Putnam, 2005; Zaman et al., 2006; Yip et al., 2015), we proceeded to determine if zyxin knockdown cells behave similarly.

**FIGURE 4:**
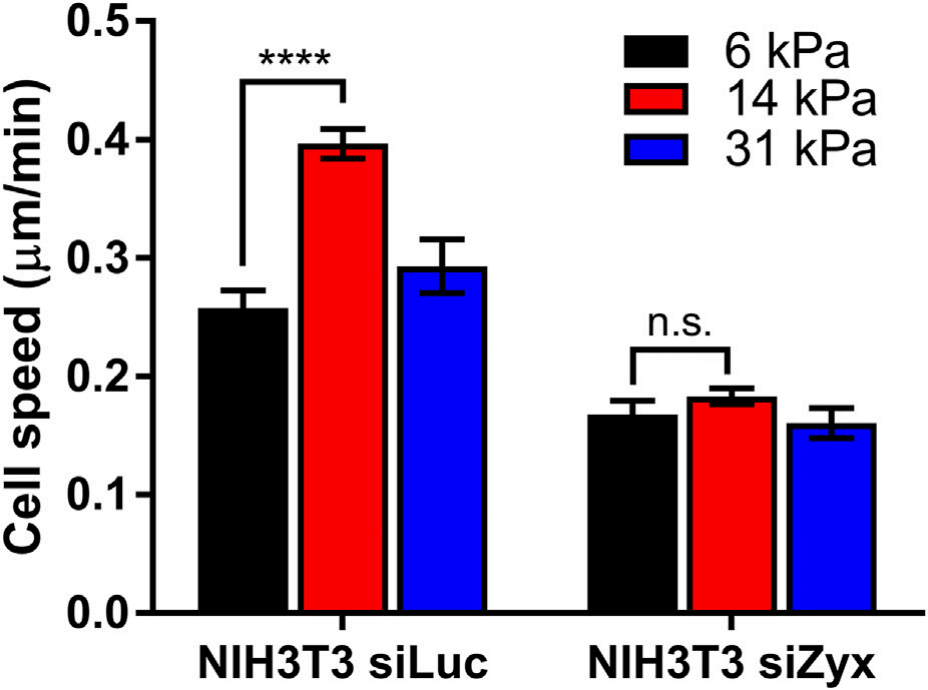
Graph of cell speed vs substrate rigidity for siLuc- and siZyx- NIH3T3 cells. n = 62, 161, and 54 for siLuc-NIH3T3 6 kPa, 14 kPa, and 31 kPa, respectively. n = 80, 81, and 85 for siZyx-, NIH3T3 6 kPa, 14 kPa, and 31 kPa respectively. Error bars represent standard error of the mean. **** represents *p* < 0.001 and n.s represents not significant.

In agreement with previous studies, control siLuc-NIH3T3 cells migrated fastest at an intermediate substrate rigidity of 14 kPa (average cell speed = 0.26 ± 0.02 μm/min, 0.40 ± 0.01 μm/min, 0.29 ± 0.02 μm/min for substrate rigidities 6 kPa, 14 and 31 kPa, respectively) (Figure 4). However, for siZyx-NIH3T3 cells, the average cell migration speed remained similar at different substrate rigidity (0.17 ± 0.01 μm/min, 0.18 ± 0.01 μm/min, and 0.16 ± 0.01 μm/min, for substrate rigidities of 6 kPa, 14 and 31 kPa, respectively).

### Fibroblasts With Zyxin Knockdown do Not Migrate Towards Regions of Increasing Substrate Rigidity

**FIGURE 5:**
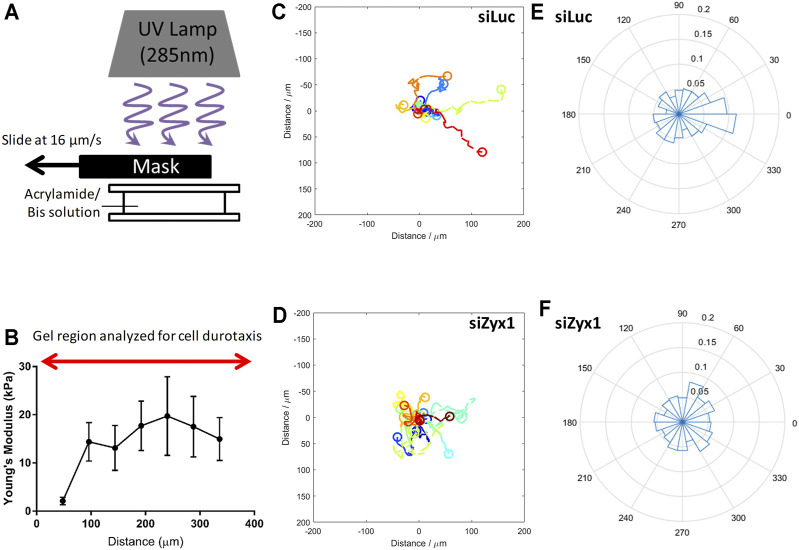
Zyxin-knockdown NIH3T3 cells do not exhibit durotaxis **(A)** Illustration of method used to fabricate gradient polyacrylamide gels **(B)** Young’s modulus of the polyacrylamide gradient gel as a function of distance. Trajectories of NIH3T3 cells transfected with **(C)** control siLuc (n = 15) and **(D)** siZyx1 siRNA (n = 16) over period of 6 h. Different colors represent the cell trajectories for each cell. Rose plot showing direction cell migration of NIH3T3 cells transfected with **(E)** control siLuc and **(F)** siZyx siRNA.

The above observations supported the hypothesis that the absence of zyxin at the FA impairs the cells’ ability to sense and respond to increasing substrate rigidity. This may subsequently lead to inability to migrate towards regions of increasing substrate rigidity during durotaxis. To test this hypothesis, we fabricated polyacrylamide gels with a gradient in substrate rigidity (Figures 5A,B) as reported in (Sunyer et al., 2012; Sunyer et al., 2016), and analyzed the migration direction of control and siZyx-NIH3T3 cells on these substrates.

We then quantified the direction of cell migration as shown in Figures 5C–F. In Figures 5C,D, the cell trajectories were presented using different colors for each cell. We then compiled all the directions of cell migration over all time points in the rose plots shown in Figures 5E,F. We took the cell displacement vector points and calculated the angle of the cell displacement vector with respect to (wrt) the *x*-axis at each timepoint for all cells. For example, 0.1 label (in the radial direction) would correspond to 10% of the cell displacement vector angle points to the 0–20° direction. Given that there were 30 timepoints, each cell contributed 30 data points in the rose plot.

As shown in Figures 5C,E (siLuc), majority of the cells were migrating toward the right (stiffer substrate) while the siZyx (Figures 5D,F) were migrating in all directions, suggesting that control NIH3T3 cells generally migrate in the direction of increasing substrate rigidity, but siZyx-NIH3T3 cells do not show such bias and exhibited random migration. Overall, these results support the hypothesis that zyxin facilitates durotaxis of fibroblasts through increasing FA sizes, cell traction stress and actin stress fibre alignment on more rigid substrates.

## Discussion

Although zyxin has been implicated as a player in mechanotransduction (Beckerle, 1986; Beckerle, 1997; Nix and Beckerle, 1997; Drees et al., 1999; Yoshigi et al., 2005; Hoffman et al., 2006; Hirata et al., 2008a; Hirata et al., 2008b), the underlying mechanism of zyxin in rigidity sensing and durotaxis has not been established. Kim and Wirtz showed that ablation of zyxin impairs cell migration (Kim and Wirtz, 2013) while Beckerle’s and Sokabe’s groups reported that mislocalization of zyxin perturbs cell spreading and cell migration (Beckerle, 1986; Beckerle, 1997; Nix and Beckerle, 1997; Drees et al., 1999; Yoshigi et al., 2005; Hoffman et al., 2006; Hirata et al., 2008a; Hirata et al., 2008b). However, it is still not clear whether zyxin might be involved in rigidity sensing that leads to directional cell migration. Taken all these together, we are motivated to investigate the role of zyxin in rigidity sensing and especially the role of zyxin in durotaxis.

In this study, we pursued the possibility that zyxin is involved in rigidity sensing. To address this possibility, we knocked down zyxin in mouse embryonic fibroblast, NIH3T3 using siRNA. We then examined the consequence of the zyxin knockdown on the levels of other key FA proteins. We found that zyxin knockdown did not affect the protein levels of talin, vinculin and paxillin. This is crucial to ensure that the observations of zyxin siRNA treatment in the following experiments are due to the depletion of zyxin and not of other FA proteins. Our study has shown that when zyxin is knocked down, both FA growth and stress fibre reorganization in response to increasing substrate rigidity were inhibited, leading to inability to increase traction stress on more rigid substrates. Loss of rigidity sensing also led to zyxin-knockdown cells migrating randomly instead of moving towards stiffer substrates. Our experimental data suggest that the loss of zyxin at the FAs impaired the substrate sensing capacity of the cells resulting in failure to exhibit durotaxis.

Previous studies have proposed that higher cell traction stress allowed cells to migrate more quickly on substrates of intermediate rigidity, while the larger cell substrate FAs slowed cell migration on substrates of high rigidity (DiMilla et al., 1991; Peyton and Putnam, 2005; Zaman et al., 2006). We have shown that since the zyxin knockdown cells do not show differences in their cell traction stress and FAs, these cells also did not exhibit changes in their cell migration speed in response to substrate rigidity. This is unlike the control NIH3T3 cells which migrated the fastest at an intermediate rigidity (14 kPa). Our findings are in concordance with a previous report by Kim and Wirtz (Kim and Wirtz, 2013), showing that focal adhesion sizes are positively correlated with substrate stiffness. Nevertheless, the substrate stiffness in our current study is not in similar range reported by Kim and Wirtz. If zyxin is depleted from the cells, the functional relationship between focal adhesion size and substrate stiffness is disrupted, and resulting in less significant changes on cell migration speed in response to substrate stiffness.

Focal adhesions have been associated to the guidance of cell migration through durotaxis (Plotnikov et al., 2012; Wu et al., 2017). While most studies elucidate the focal adhesion complex as a whole in rigidity sensing to mediate durotaxis (Kim and Wirtz, 2013), the roles of individual FA proteins are not well studied in this context. The involvement of focal adhesion kinase (FAK) in mechanosensing and durotaxis has been reported (Wang et al., 2001)^.^ In addition to FAK, vinculin and talin also mediate substrate sensing (Wu et al., 2017), although their direct roles in durotaxis remain to be explored. In another study, cdGAP, an adhesion-localized Rac1 and Cdc42 specific GTPase-activating protein, has been reported to regulate durotaxis (Wormer et al., 2014). Our study further fills in the gap to uncover new insights on the roles of zyxin in rigidity sensing and durotaxis. Future work will include integration of the independent roles exerted by major focal adhesion components and to determine how these focal adhesion proteins work cooperatively in time and space to regulate durotaxis.

## Conclusion

**FIGURE 6:**
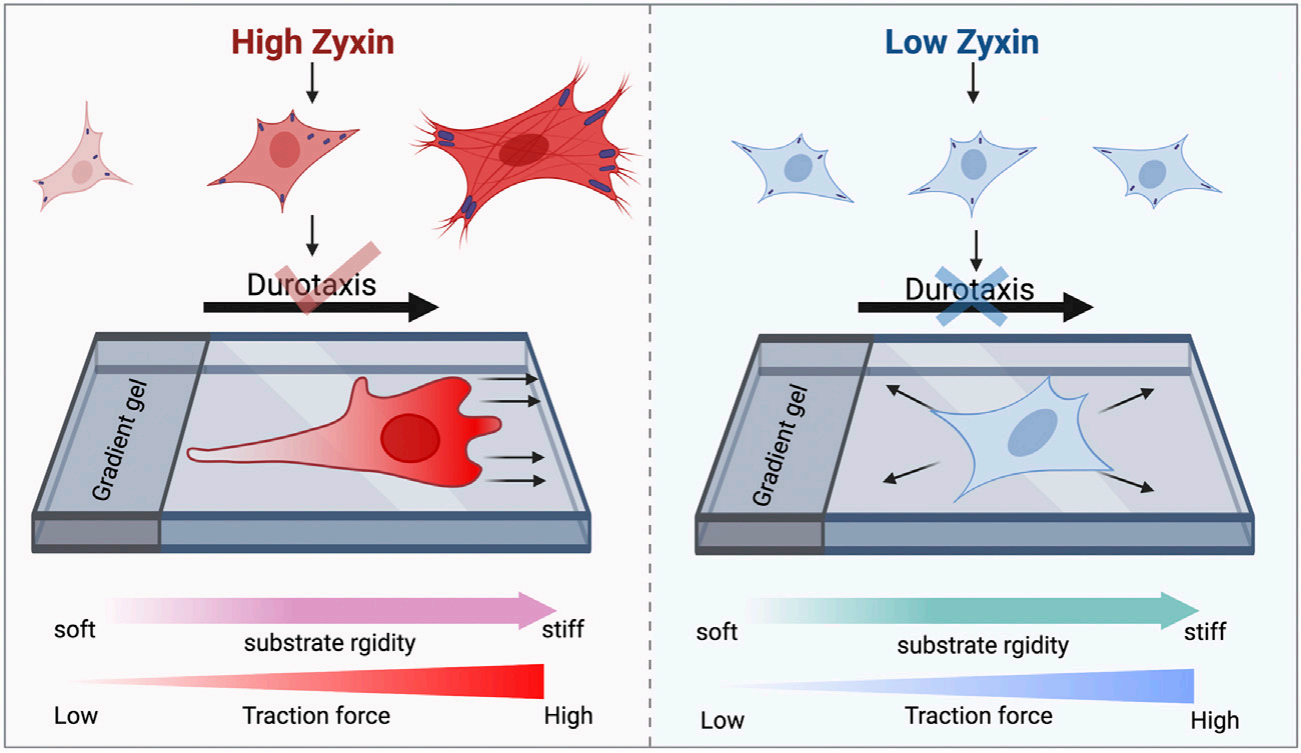
Zyxin facilitates substrate rigidity sensing and impacts on durotaxis. Cells with high zyxin levels can sense substrate rigidity through modulating actin, focal adhesion, and traction stress, allowing the cells to move up rigidity gradients **(left panel)**. Cells with low zyxin levels **(right panel)** are not able to sense substrate rigidity because they have lost the ability to tune cellular actin, focal adhesion, and traction stress in response to changes on substrate stiffness. Therefore, these cells are not able to perform durotaxis and they migrate randomly in all directions.

As shown in Figure 6, cells with high zyxin levels can sense substrate rigidity through modulating actin, focal adhesion, and traction stress, allowing the cells to move towards to the stiffer substrate (left panel). Cells with low zyxin levels are not able to sense substrate rigidity because they have lost the ability to tune cellular actin, focal adhesion, and traction stress in response to changes on substrate stiffness. Therefore, these cells are not able to perform durotaxis and they migrate randomly in all directions. Our observations highlight the significance of zyxin as one of the possible focal adhesion components involved in substrate rigidity sensing. Without rigidity sensing, the zyxin-knockdown fibroblasts also lose their ability to undergo durotaxis.

## Data Availability

The raw data supporting the conclusions of this article will be made available by the authors, without undue reservation.

## References

[B1] Beckerle M. C. (1986). Identification of a New Protein Localized at Sites of Cell-Substrate Adhesion. J. Cel Biol 103 (5), 1679–1687. 10.1083/jcb.103.5.1679 PMC2114371 3536951

[B2] Beckerle M. C. (1997). Zyxin: Zinc Fingers at Sites of Cell Adhesion. Bioessays 19 (11), 949–957. 10.1002/bies.950191104 9394617

[B3] Califano J. P. Reinhart-King C. A. (2010). Substrate Stiffness and Cell Area Predict Cellular Traction Stresses in Single Cells and Cells in Contact. Cel. Mol. Bioeng. 3 (1), 68–75. 10.1007/s12195-010-0102-6 PMC2992361 21116436

[B4] DiMilla P. A. Barbee K. Lauffenburger D. A. (1991). Mathematical Model for the Effects of Adhesion and Mechanics on Cell Migration Speed. Biophysical J. 60 (1), 15–37. 10.1016/s0006-3495(91)82027-6 PMC1260035 1883934

[B5] Discher D. E. Janmey P. Wang Y.-l. (2005). Tissue Cells Feel and Respond to the Stiffness of Their Substrate. Science 310 (5751), 1139–1143. 10.1126/science.1116995 16293750

[B6] Drees B. E. Andrews K. M. Beckerle M. C. (1999). Molecular Dissection of Zyxin Function Reveals its Involvement in Cell Motility. J. Cel Biol 147 (7), 1549–1560. 10.1083/jcb.147.7.1549 PMC2174240 10613911

[B7] Franck C. Maskarinec S. A. Tirrell D. A. Ravichandran G. (2011). Three-Dimensional Traction Force Microscopy: A New Tool for Quantifying Cell-Matrix Interactions. PLOS ONE 6 (3), e17833. 10.1371/journal.pone.0017833 21468318 PMC3066163

[B8] Harborth J. Elbashir S. M. Bechert K. Tuschl T. Weber K. (2001). Identification of Essential Genes in Cultured Mammalian Cells Using Small Interfering RNAs. J. Cel Sci 114 (Pt 24), 4557–4565. 10.1242/jcs.114.24.4557 11792820

[B9] Hirata H. Tatsumi H. Sokabe M. (2008). Mechanical Forces Facilitate Actin Polymerization at Focal Adhesions in a Zyxin-dependent Manner. J. Cel Sci. 121 (17), 2795–2804. 10.1242/jcs.030320 18682496

[B10] Hirata H. Tatsumi H. Sokabe M. (2008). Zyxin Emerges as a Key Player in the Mechanotransduction at Cell Adhesive Structures. Communicative Integr. Biol. 1 (2), 192–195. 10.4161/cib.1.2.7001 PMC2686020 19513257

[B11] Hoffman L. M. Jensen C. C. Kloeker S. Wang C.-L. A. Yoshigi M. Beckerle M. C. (2006). Genetic Ablation of Zyxin Causes Mena/VASP Mislocalization, Increased Motility, and Deficits in Actin Remodeling. J. Cel Biol 172 (5), 771–782. 10.1083/jcb.200512115 PMC2063708 16505170

[B12] Hu Y.-L. Lu S. Szeto K. W. Sun J. Wang Y. Lasheras J. C. (2014). FAK and Paxillin Dynamics at Focal Adhesions in the Protrusions of Migrating Cells. Sci. Rep. 4 (1), 6024. 10.1038/srep06024 25113375 PMC4129417

[B13] Kanchanawong P. Shtengel G. Pasapera A. M. Ramko E. B. Davidson M. W. Hess H. F. (2010). Nanoscale Architecture of Integrin-Based Cell Adhesions. Nature 468 (7323), 580–584. 10.1038/nature09621 21107430 PMC3046339

[B14] Kim D. H. Wirtz D. (2013). Focal Adhesion Size Uniquely Predicts Cell Migration. FASEB j. 27 (4), 1351–1361. 10.1096/fj.12-220160 23254340 PMC3606534

[B15] Kobayashi T. Sokabe M. (2010). Sensing Substrate Rigidity by Mechanosensitive Ion Channels with Stress Fibers and Focal Adhesions. Curr. Opin. Cel Biol. 22 (5), 669–676. 10.1016/j.ceb.2010.08.023 20850289

[B16] Legerstee K. Geverts B. Slotman J. A. Houtsmuller A. B. (2019). Dynamics and Distribution of Paxillin, Vinculin, Zyxin and VASP Depend on Focal Adhesion Location and Orientation. Sci. Rep. 9 (1), 10460. 10.1038/s41598-019-46905-2 31320676 PMC6639384

[B17] Lo C.-M. Wang H.-B. Dembo M. Wang Y.-l. (2000). Cell Movement Is Guided by the Rigidity of the Substrate. Biophysical J. 79 (1), 144–152. 10.1016/S0006-3495(00)76279-5 PMC1300921 10866943

[B18] López-Colomé A. M. Lee-Rivera I. Benavides-Hidalgo R. López E. (2017). Paxillin: a Crossroad in Pathological Cell Migration. J. Hematol. Oncol. 10 (1), 50. 10.1186/s13045-017-0418-y 28214467 PMC5316197

[B19] Maskarinec S. A. Franck C. Tirrell D. A. Ravichandran G. (2009). Quantifying Cellular Traction Forces in Three Dimensions. Pnas 106 (52), 22108–22113. 10.1073/pnas.0904565106 20018765 PMC2799761

[B20] Nix D. A. Beckerle M. C. (1997). Nuclear-cytoplasmic Shuttling of the Focal Contact Protein, Zyxin: a Potential Mechanism for Communication between Sites of Cell Adhesion and the Nucleus. J. Cel Biol 138 (5), 1139–1147. 10.1083/jcb.138.5.1139 PMC2136768 9281590

[B21] Peyton S. R. Putnam A. J. (2005). Extracellular Matrix Rigidity Governs Smooth Muscle Cell Motility in a Biphasic Fashion. J. Cel. Physiol. 204 (1), 198–209. 10.1002/jcp.20274 15669099

[B22] Plotnikov S. V. Pasapera A. M. Sabass B. Waterman C. M. (2012). Force Fluctuations within Focal Adhesions Mediate ECM-Rigidity Sensing to Guide Directed Cell Migration. Cell 151 (7), 1513–1527. 10.1016/j.cell.2012.11.034 23260139 PMC3821979

[B23] Prager-Khoutorsky M. Lichtenstein A. Krishnan R. Rajendran K. Mayo A. Kam Z. (2011). Fibroblast Polarization Is a Matrix-rigidity-dependent Process Controlled by Focal Adhesion Mechanosensing. Nat. Cel Biol 13 (12), 1457–1465. 10.1038/ncb2370 22081092

[B24] Riveline D. Zamir E. Balaban N. Q. Schwarz U. S. Ishizaki T. Narumiya S. (2001). Focal Contacts as Mechanosensors. J. Cel Biol 153 (6), 1175–1186. 10.1083/jcb.153.6.1175 PMC2192034 11402062

[B25] Solon J. Levental I. Sengupta K. Georges P. C. Janmey P. A. (2007). Fibroblast Adaptation and Stiffness Matching to Soft Elastic Substrates. Biophysical J. 93 (12), 4453–4461. 10.1529/biophysj.106.101386 PMC2098710 18045965

[B26] Sunyer R. Conte V. Escribano J. Elosegui-Artola A. Labernadie A. Valon L. (2016). Collective Cell Durotaxis Emerges from Long-Range Intercellular Force Transmission. Science 353 (6304), 1157–1161. 10.1126/science.aaf7119 27609894

[B27] Sunyer R. Jin A. J. Nossal R. Sackett D. L. (2012). Fabrication of Hydrogels with Steep Stiffness Gradients for Studying Cell Mechanical Response. PLOS ONE 7 (10), e46107. 10.1371/journal.pone.0046107 23056241 PMC3464269

[B28] Trichet L. Le Digabel J. Hawkins R. J. Vedula S. R. K. Gupta M. Ribrault C. (2012). Evidence of a Large-Scale Mechanosensing Mechanism for Cellular Adaptation to Substrate Stiffness. Proc. Natl. Acad. Sci. 109 (18), 6933–6938. 10.1073/pnas.1117810109 22509005 PMC3344951

[B29] Wang H.-B. Dembo M. Hanks S. K. Wang Y.-l. (2001). Focal Adhesion Kinase Is Involved in Mechanosensing during Fibroblast Migration. Proc. Natl. Acad. Sci. 98 (20), 11295–11300. 10.1073/pnas.201201198 11572981 PMC58723

[B30] Wang N. Butler J. P. Ingber D. E. (1993). Mechanotransduction across the Cell Surface and through the Cytoskeleton. Science 260 (5111), 1124–1127. 10.1126/science.7684161 7684161

[B31] Wormer D. B. Davis K. A. Henderson J. H. Turner C. E. (2014). The Focal Adhesion-Localized CdGAP Regulates Matrix Rigidity Sensing and Durotaxis. PLOS ONE 9 (3), e91815. 10.1371/journal.pone.0091815 24632816 PMC3954768

[B32] Wu Z. Plotnikov S. V. Moalim A. Y. Waterman C. M. Liu J. (2017). Two Distinct Actin Networks Mediate Traction Oscillations to Confer Focal Adhesion Mechanosensing. Biophysical J. 112 (4), 780–794. 10.1016/j.bpj.2016.12.035 PMC5340160 28256237

[B33] Yamamura M. Noguchi K. Nakano Y. Segawa E. Zushi Y. Takaoka K. (2013). Functional Analysis of Zyxin in Cell Migration and Invasive Potential of Oral Squamous Cell Carcinoma Cells. Int. J. Oncol. 42 (3), 873–880. 10.3892/ijo.2013.1761 23292068

[B34] Yang H. Cheam N. M. J. Cao H. Lee M. K. H. Sze S. K. Tan N. S. (2019). Materials Stiffness‐Dependent Redox Metabolic Reprogramming of Mesenchymal Stem Cells for Secretome‐Based Therapeutic Angiogenesis. Adv. Healthc. Mater. 8 (20), 1900929. 10.1002/adhm.201900929 31532923

[B35] Yang H. Nguyen K. T. Leong D. T. Tan N. S. Tay C. Y. (2016). Soft Material Approach to Induce Oxidative Stress in Mesenchymal Stem Cells for Functional Tissue Repair. ACS Appl. Mater. Inter. 8 (40), 26591–26599. 10.1021/acsami.6b09222 27608498

[B36] Yip A. K. Chiam K.-H. Matsudaira P. (2015). Traction Stress Analysis and Modeling Reveal that Amoeboid Migration in Confined Spaces Is Accompanied by Expansive Forces and Requires the Structural Integrity of the Membrane-Cortex Interactions. Integr. Biol. 7 (10), 1196–1211. 10.1039/c4ib00245h 26050549

[B37] Yip A. K. Iwasaki K. Ursekar C. Machiyama H. Saxena M. Chen H. (2013). Cellular Response to Substrate Rigidity Is Governed by Either Stress or Strain. Biophysical J. 104 (1), 19–29. 10.1016/j.bpj.2012.11.3805 PMC3540269 23332055

[B38] Yoshigi M. Hoffman L. M. Jensen C. C. Yost H. J. Beckerle M. C. (2005). Mechanical Force Mobilizes Zyxin from Focal Adhesions to Actin Filaments and Regulates Cytoskeletal Reinforcement. J. Cel Biol 171 (2), 209–215. 10.1083/jcb.200505018 PMC2171187 16247023

[B39] Zaman M. H. Trapani L. M. Sieminski A. L. MacKellar D. Gong H. Kamm R. D. (2006). Migration of Tumor Cells in 3D Matrices Is Governed by Matrix Stiffness along with Cell-Matrix Adhesion and Proteolysis. Proc. Natl. Acad. Sci. 103 (29), 10889–10894. 10.1073/pnas.0604460103 16832052 PMC1544144

