# Supplementary figures and images for: Zyxin Is Involved in Fibroblast Rigidity Sensing and Durotaxis
Source: Front Cell Dev Biol. 2021 Nov 18;9:735298. doi: 10.3389/fcell.2021.735298 (PMC8637444; doi:10.3389/fcell.2021.735298)

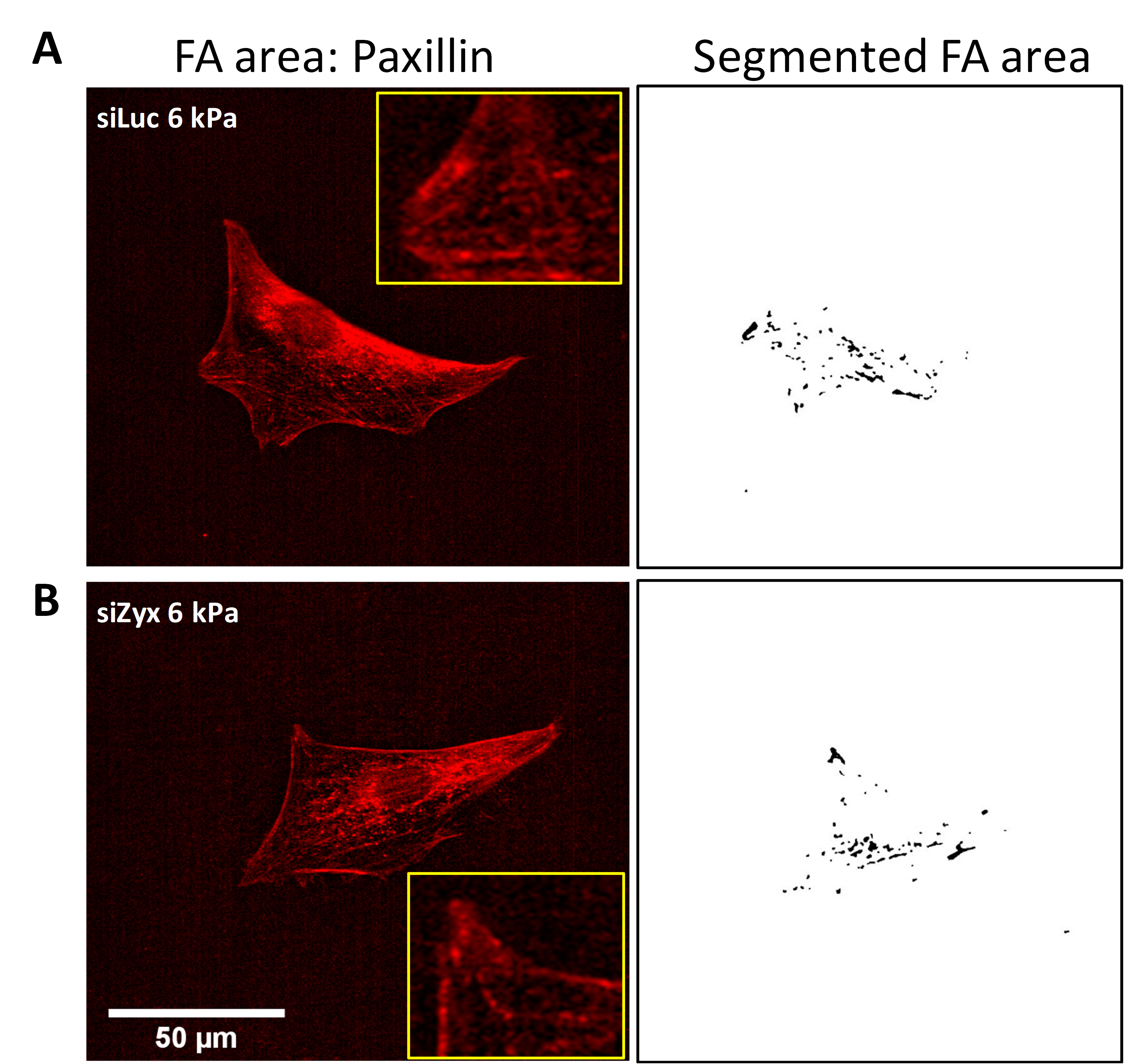

Supplement: Supplementary file 1 [file Image1.jpeg]

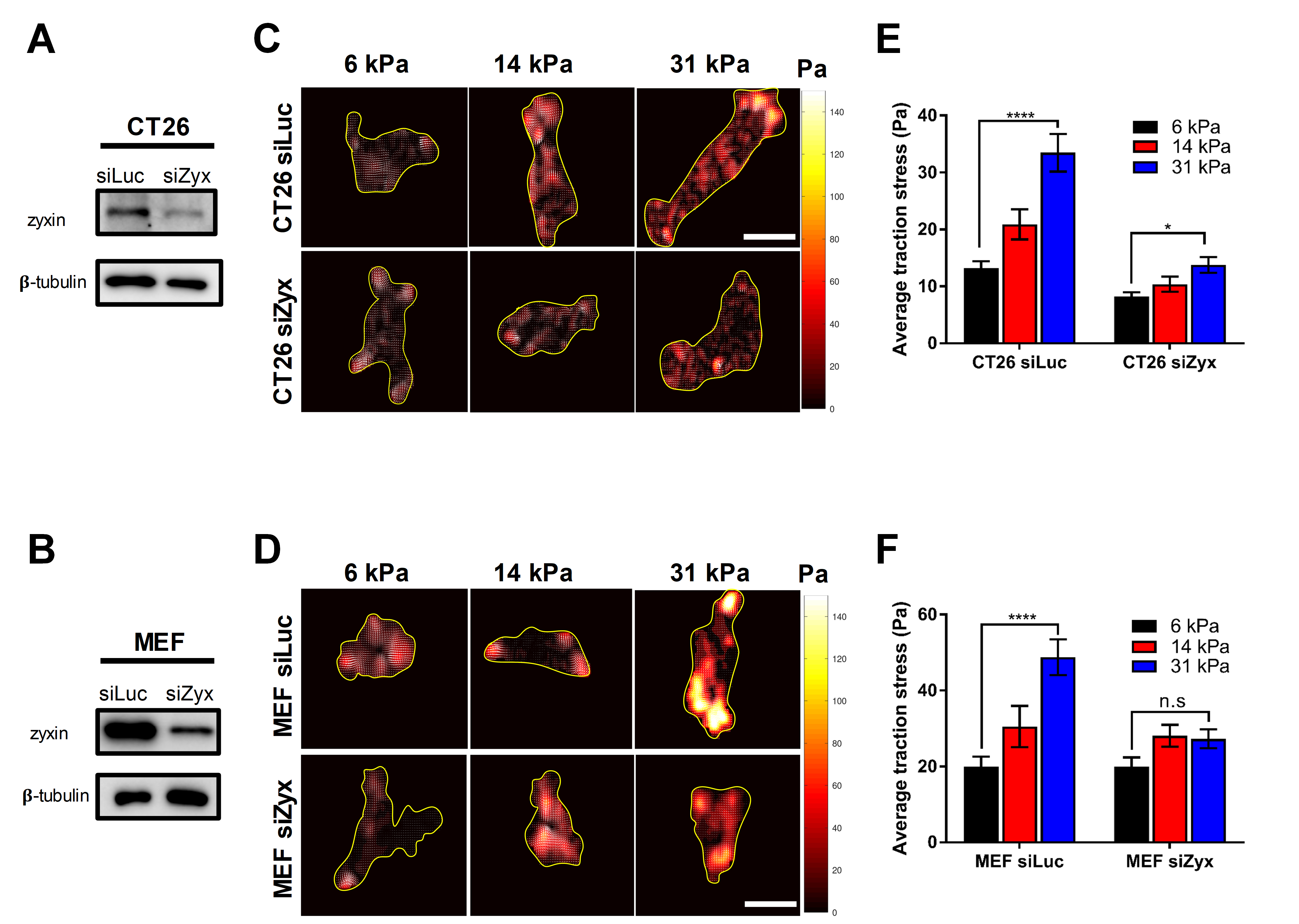

Supplement: Supplementary file 2 [file Image2.jpeg]
